# Jump and Pull-in Instability of a MEMS Gyroscope Vibrating System

**DOI:** 10.3390/mi14071396

**Published:** 2023-07-08

**Authors:** Yijun Zhu, Huilin Shang

**Affiliations:** School of Mechanical Engineering, Shanghai Institute of Technology, Shanghai 201418, China

**Keywords:** MEMS gyroscope, bistability, jump, pull-in instability, saddle-node bifurcation, heteroclinic bifurcation, fractal

## Abstract

Jump and pull-in instability are common nonlinear dynamic behaviors leading to the loss of the performance reliability and structural safety of electrostatic micro gyroscopes. To achieve a better understanding of these initial-sensitive phenomena, the dynamics of a micro gyroscope system considering the nonlinearities of the stiffness and electrostatic forces are explored from a global perspective. Static and dynamic analyses of the system are performed to estimate the threshold of the detecting voltage for static pull-in, and dynamic responses are analyzed in the driving and detecting modes for the case of primary resonance and 1:1 internal resonance. The results show that, when the driving voltage frequency is a bit higher than the natural frequency, a high amplitude of the driving AC voltage may induce the coexistence of bistable periodic responses due to saddle-node bifurcation of the periodic solution. Basins of attraction of bistable attractors provide evidence that disturbance of the initial conditions can trigger a jump between bistable attractors. Moreover, the Melnikov method is applied to discuss the condition for pull-in instability, which can be ascribed to heteroclinic bifurcation. The validity of the prediction is verified using the sequences of safe basins and unsafe zones for dynamic pull-in. It follows that pull-in instability can be caused and aggravated by the increase in the amplitude of the driving AC voltage.

## 1. Introduction

Micromachined vibratory gyroscopes are typical MEMS inertia sensors used for the measurement of the angular velocities of carriers [1,2]. Their operation is based on energy transfer from the driving mode to its perpendicular vibrational mode, i.e., the detecting mode, due to Coriolis effect [3]. The magnitude of oscillation in the detecting mode provides a measure of the detected angular velocity. The electrostatic principles are the most widely used actuation and transduction principles of micro gyroscopes [4]. To achieve a high operating performance, electrostatic micro gyroscopes are supposed to work in a linear state and provide a linear response. However, due to the microscale effect, there are inherent nonlinear factors in the vibratory systems of electrostatic micro gyroscopes that stem from damping [5], flexure springs [6] and electrostatic forces [7]. It usually leads to nonlinear dynamic behaviors and the loss of stability, sensitivity and reliability of the micro gyroscopes. 

To improve the performance and exploit the application of electrostatic micro gyroscopes, there have been a few studies concerning their nonlinear dynamics in recent years. The characteristics of harden/soften nonlinear stiffness was found in 2-DOF lumped-parameter vibrating systems of electrostatic micro gyroscopes [8,9,10,11,12,13]. Kacem [8] proposed a Mathieu resonator for micro gyroscope applications and established its practical design on the basis of the analytical study of its nonlinear dynamics. Considering a micromechanical gyroscope excited by the driving electrostatic torque, Awrejcewicz [9] presented the approximate solution of its dynamic system analytically under the simultaneous occurrence of the main and internal resonances. Zhang et al. [10] investigated the local bifurcation of the periodic response of a micro gyroscope with cubic supporting stiffness and fractional electrostatic forces and found the parameter regions to meet the performance requirements of the micro sensor. For a 4-DOF micro gyroscope system with nonlinearity of the driving stiffness, primary resonance and 1:1 internal resonance, the spring hardening effect as well as multistability were also observed [14]. 

For the normal work performance of micro gyroscopes, multistability is undesirable, as a jump among multiple responses can lead to the loss of the detection reliability of the devices. Oropeza-Ramos et al. [15] presented the jump phenomenon of the driving mode of a novel Micro-electro gyroscope system experimentally. Shang et al. [16] depicted the jump between bistable periodic responses quantitatively via the classification of the basins of attraction. Nevertheless, there are few studies concerning the effect of the initial conditions on inducing the jump of micro gyroscopes, because in most previous theoretical studies on jumps, the disturbance of the initial conditions was ignored; namely, the initial conditions were tacitly assumed to be unchanged.

Complex dynamic behaviors, such as quasi-periodic responses [17] and chaos [18,19], were also found in the vibration of MEMS electrostatic gyroscopes. Tsai [18] focused on the resonance of a MEMS ring gyroscope under nonlinearity effects and numerically inspected the chaotic behavior of the driving and sensing modes. 

Another initial-sensitive phenomenon of electrostatic micro gyroscopes is pull-in instability, which is closely related to the pull-in behavior. The pull-in of capacitors means the contact of two neighboring capacitors and the stop of micro gyroscopes’ operation. To be specific, there are two types of pull-in: static pull-in [20,21,22] and dynamic pull-in [23,24]. The former comes from the imbalanced static case of the microstructures, thus being unavoidable, whereas the occurrence of the latter relies on the initial states of the oscillatory systems. For instance, Ouakad [23] numerically displayed the dynamic pull-in caused by variations in the static displacement of a vibrating-beam micro gyroscope at its connected tip. Pull-in instability represents the sensitivity of the initial conditions, leading to dynamic pull-in. In other words, a small disturbance of the initial conditions induces a sudden pull-in, meaning the loss of the structural safety of the micro devices. In single-degree-of-freedom micro resonator systems, pull-in instability has been extensively studied [25,26,27]. However, in multiple-degree-of-freedom micro gyroscope systems, the mechanism of the pull-in instability of capacitors has not yet been studied.

Therefore, in this study, two initial-sensitive phenomena of the MEMS gyroscopes, namely jump and pull-in instability, are investigated. The outline of this paper is as follows: First, the vibrating system of a typical electrostatic micro gyroscope and its unperturbed system are proposed; on this basis, the voltage threshold for the static pull-in of the detecting direction is analyzed. In Section 3, via the analysis of the local bifurcation of periodic solutions and the classification of basins of attraction, conditions for the coexistence of multiple responses and jumps are presented. Then, the phenomenon of pull-in instability is discussed analytically and numerically in Section 4. Finally, Section 5 and Section 6 contain the discussion and conclusions.

## 2. Dynamic Model of a MEMS Gyroscope and Its Unperturbed System

**Figure 1:**
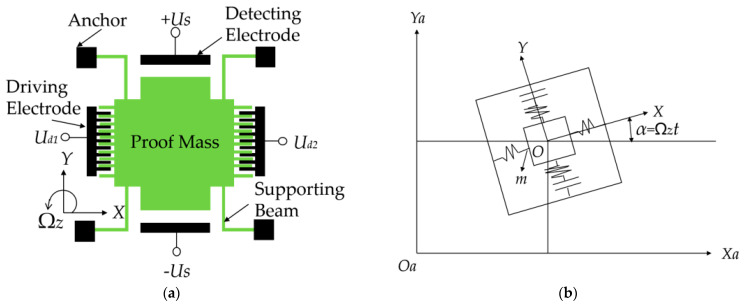
Model of a comb-tooth micro gyroscope: (**a**) Schematic representation of the micro gyroscope; (**b**) Equivalent spring-mass model of the micro gyroscope.

We consider a typical comb-tooth micro-machined gyroscope driven by electrostatic forces, whose schematic representation is shown in Figure 1 [10,15,16]. Here, *m* represents the sensitive mass of the micro gyroscope physically existing in two operating modes, i.e., the driving mode and the detecting mode. *X_a_*-*O_a_-Y_a_* means the absolute coordinate system, where $X_{a}$ and $Y_{a}$ are the horizontal and vertical displacements of the sensitive mass, respectively, whereas *XOY* means the relative coordinate system of the driving and detecting modes of the micro gyroscope, where the origin O is the center of the sensitive mass and *X* and *Y* are the relative displacements of the mass in the driving and detecting directions at moment *t,* respectively. The Z axis is perpendicular to the plane *XOY*. α is the angular displacement of the gyroscope around the *z*-axis. $\Omega_{z}$ is the angular velocity of the carrier of the MEMS gyroscope to be measured. As can be seen in Figure 1, the micro gyroscope is actuated by electrostatic forces. The detecting voltage is DC voltage, noted by $U_{S}$. In the driving direction, voltages $U_{d1}$ and $U_{d2}$ can be expressed by [10,15]

(1) $$U_{d1}=U_{d0}+U_{da}\cos\Omega t,\;U_{d2}=U_{d0}-U_{da}\cos\Omega t,$$

where $U_{d0}$ is the bias DC voltage and $U_{da}$ and Ω are the amplitude and frequency of the AC voltage, respectively.

In the vacuum environment of this micro-gyroscope, air damping is related to the degree of the vacuum, which means that the air damping is relatively small, and its nonlinear factors can be ignored. The nonlinearities of the stiffness of the driving and detecting modes are taken into account. The electrostatic forces in the driving and detecting directions are also nonlinear, as they are nonlinear functions of the driven voltages. Considering these factors and based on the Lagrange equations, the vibrating system of the sensitive mass can be expressed as the following 2-DOF dynamic equation:

(2) $$\begin{array}{l} m\ddot{X}+c_{X}\dot{X}+k_{X1}X+k_{X2}X^{3}-2m\Omega_{z}\dot{Y}=F_{X}\cos\Omega t-ma_{X},\\ m\ddot{Y}+c_{Y}\dot{Y}+k_{Y1}Y+k_{Y2}Y^{3}+2m\Omega_{z}\dot{X}=\frac{F_{Y}Y}{{\left(d_{eg}-Y\right)}^{2}{\left(d_{eg}+Y\right)}^{2}}-ma_{Y}. \end{array}$$

where the electrostatic forces $F_{X}$ and $F_{Y}$ can be given by [10,15]

(3) $$F_{X}=\frac{4n\zeta aU_{d0}U_{da}}{s_{d}},\;F_{Y}=2\zeta l_{l}l_{w}d_{eg}U_{s}^{2},$$

The physical interpretation of the parameters in Equations (2) and (3) is provided in Table 1. Here, the device is considered to be made of polysilicon, relaxed at room temperature (300 K) and in a high vacuum environment. As the validity of this model was examined extensively in [15], the values of the geometrical and material parameters in the following calculations are adopted from [15] (see Table 1). Without detriment to accuracy, this model is able to properly catch the essential aspects of the nonlinearities of micro gyroscopes. Referring to it, we focus on the effect of the AC voltage frequency Ω and amplitude $U_{da}$ in the drive direction on the global dynamics of the micro gyroscope. Note that $Y=\pm d_{eg}$ in Equation (2) shows the gap width between the proof mass and the upper or lower movable electrode of the detecting direction becoming zero, namely the pull-in of the micro gyroscope in the detecting direction.

By introducing the following non-dimensional variables

(4) $$\begin{array}{l} x=\frac{X}{d_{eg}},y=\frac{Y}{d_{eg}},\omega_{0}=\sqrt{\frac{k_{Y1}}{m}},T=\omega_{0}t,\omega =\frac{\Omega }{\omega_{0}},\dot{x}=\frac{dx}{dT},\mu_{1}=\frac{c_{X}}{m\omega_{0}},\mu_{2}=\frac{c_{Y}}{m\omega_{0}},\omega_{x}{}^{2}=\frac{k_{X1}}{m\omega_{0}{}^{2}},\\ k_{1}=\frac{k_{X2}d_{eg}{}^{2}}{m\omega_{0}{}^{2}},k_{2}=\frac{k_{Y2}d_{eg}{}^{2}}{m\omega_{0}{}^{2}},\eta =\frac{\Omega_{z}}{\omega_{0}},f=\frac{4n\zeta d_{a}U_{d0}U_{da}}{m\omega_{0}{}^{2}d_{eg}s_{d}},\beta =\frac{2\zeta l_{l}l_{w}U_{s}^{2}}{m\omega_{0}{}^{2}d_{eg}{}^{3}},A_{x}=\frac{a_{X}}{\omega_{0}{}^{2}d_{eg}},A_{y}=\frac{a_{Y}}{\omega_{0}{}^{2}d_{eg}}. \end{array}$$

into Equation (2), we can obtain the following non-dimensional system:

(5) $$\begin{array}{l} \frac{d^{2}x}{dT^{2}}+\mu_{1}\frac{dx}{dT}+\omega_{x}{}^{2}x+k_{1}x^{3}-2\eta \frac{dy}{dT}=f\cos\omega T-A_{x},\\ \frac{d^{2}y}{dT^{2}}+\mu_{2}\frac{dy}{dT}+y+k_{2}y^{3}+2\eta \frac{dx}{dT}=\frac{\beta y}{{\left(1-y^{2}\right)}^{2}}. \end{array}$$

All non-dimensional parameters in the above equation are positive. Variable *x* in Equation (5) should satisfy |y| ≤ 1. |y| = 1 means the pull-in of the microstructures in the detecting direction.

The equilibria of system (5) can be solved using the following equation:

(6) $$\omega_{x}{}^{2}x+k_{1}x^{3}=-A_{x},\;y+k_{2}y^{3}=\frac{\beta y}{{\left(1-y^{2}\right)}^{2}}.$$

The static bifurcation of the equilibria was discussed in [27], according to which β=1 is the fork–bifurcation point. There are two cases for the number of equilibria and the potential well of system (5), as follows:

1. For $2\geq k_{2}\geq 0,\;\beta \geq 1$ or $k_{2}\geq 2,\;\beta \geq \frac{4{\left(1+k_{2}\right)}^{3}}{27k_{2}^{2}}$, there is only one equilibrium and no potential well, meaning that all unperturbed orbits are unbounded. In the detecting direction, the unbounded orbits inevitably lead to pull-in. Returning the conditions of parameters $k_{2}$ and *β* to the original system’s parameters, we can obtain the conditions of the detecting voltage for static pull-in:
  (7) $$U_{S}\geq \sqrt{\frac{k_{Y1}d_{eg}{}^{3}}{2\zeta l_{l}l_{w}}}\;\mathrm{in}\;\mathrm{the}\;\mathrm{case}\;\mathrm{of}\;2\geq \frac{k_{Y2}d_{eg}{}^{2}}{k_{Y1}}\geq 0,$$
  or
  (8) $$U_{S}\geq \frac{\sqrt{2}{\left(k_{Y1}+k_{Y2}d_{eg}{}^{2}\right)}^{\frac{3}{2}}}{3k_{Y2}\sqrt{3\zeta l_{l}l_{w}d_{eg}}}\;\mathrm{in}\;\mathrm{the}\;\mathrm{case}\;\mathrm{of}\;\frac{k_{Y2}d_{eg}{}^{2}}{k_{Y1}}\geq 2.$$
2. For 0<β<1, namely
  (9) $$0<U_{S}<\sqrt{\frac{k_{X1}d_{eg}{}^{3}}{2\zeta l_{l}l_{w}}}$$
  there are three equilibria of system (5). One equilibrium, C $\left(x_{c},0,0,0\right)$, is the center of a potential well surrounded by heteroclinic orbits crossing two saddles, $S_{\pm }(x_{c},0,\pm y_{s},0)$, where
  (10) $$\omega_{x}{}^{2}x_{c}+k_{1}x_{c}{}^{3}=-A_{x},\;1+k_{2}y_{s}{}^{2}=\frac{\beta }{{\left(1-y_{s}{}^{2}\right)}^{2}},\;y_{s}>0.$$

For case 2, to determine the exact heteroclinic orbits, we let $x=x_{c}+\tilde{x}$ to translate the origin O to the center C $\left(x_{c},0,0,0\right)$ and to rewrite the nondimensional system (5) as

(11) $$\begin{array}{l} \frac{d^{2}\tilde{x}}{dT^{2}}+\omega_{x}{}^{2}\tilde{x}=-\mu_{1}\frac{d\tilde{x}}{dT}-k_{1}(3x_{c}{}^{2}\tilde{x}+3x_{c}{\tilde{x}}^{2}+{\tilde{x}}^{3})+\eta \frac{dy}{dT}+f\cos\omega T,\\ \frac{d^{2}y}{dT^{2}}+y+k_{2}y^{3}-\frac{\beta y}{{\left(1-y^{2}\right)}^{2}}=-\mu_{2}\frac{dy}{dT}-\eta \frac{d\tilde{x}}{dT}. \end{array}$$

Its unperturbed system and Hamiltonian function can be expressed as

(12) $$\frac{d\tilde{x}}{dT}=u,\;\frac{du}{dT}=-\omega_{x}{}^{2}\tilde{x},\;\frac{dy}{dT}=v,\;\frac{dv}{dT}=-y-k_{2}y^{3}+\frac{\beta y}{{\left(1-y^{2}\right)}^{2}}.$$

and

(13) $$H(\tilde{x},u,y,v)=\frac{1}{2}(\omega_{x}{}^{2}{\tilde{x}}^{2}+u^{2}+y^{2}+\frac{k_{2}y^{4}}{2}-\frac{\beta }{1-y^{2}}+\beta +v^{2}),$$

respectively. Hence, the corresponding heteroclinic orbits (${\tilde{x}}_{h},u_{h},y_{h},v_{h}$) can be given by

(14) $$\omega_{x}{}^{2}{\tilde{x}}_{h}{}^{2}+u_{h}{}^{2}+y_{h}{}^{2}+\frac{k_{2}y_{h}{}^{4}}{2}-\frac{\beta }{1-y_{h}{}^{2}}+v_{h}{}^{2}=y_{s}{}^{2}+\frac{k_{2}y_{s}{}^{4}}{2}-\frac{\beta }{1-y_{s}{}^{2}}.$$

Obviously, the unperturbed system (12) is a high-dimensional system whose heteroclinic orbits are in a four-dimensional space, thus being hard to depict. Based on the unperturbed system (12), we introduce a new variable, R, to express ${\tilde{x}}_{h}$ and $u_{h}$, as shown below:

(15) $${\tilde{x}}_{h}=\frac{R}{\omega_{x}}\cos(\omega_{x}T),\;u_{h}=-R\sin(\omega_{x}T),$$

Then, the heteroclinic orbits can be depicted in the three-dimensional space (R,y,v), given by

(16) $$R^{2}+y_{h}{}^{2}+\frac{k_{2}y_{h}{}^{4}}{2}-\frac{\beta }{1-y_{h}{}^{2}}+v_{h}{}^{2}=y_{s}{}^{2}+\frac{k_{2}y_{s}{}^{4}}{2}-\frac{\beta }{1-y_{s}{}^{2}}.$$

According to Table 1, the invariable parameters of the non-dimensional system (11) can be calculated as

(17) $$\omega_{x}=0.9747,\;k_{1}=0.05,\;k_{2}=0.05,\;\beta =0.04,\;A_{x}=0.4,\;\mu_{1}=0.015,\;\mu_{2}=0.015,\;\eta =0.01.$$

**Figure 2:**
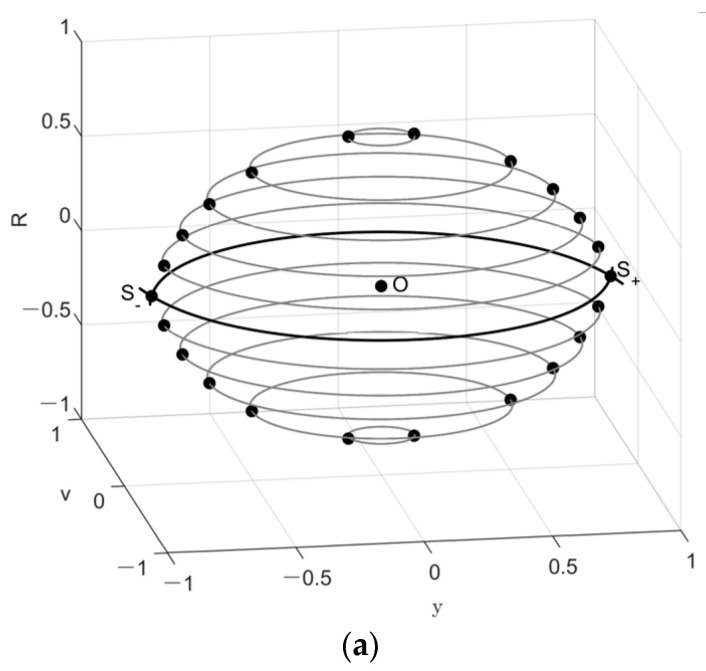

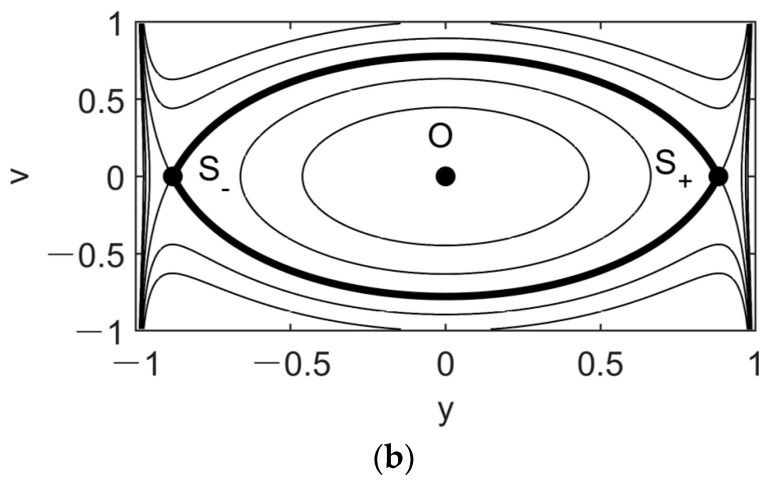
Orbits of the unperturbed system (5): (**a**) Heteroclinic orbits in three-dimensional space; (**b**) Orbits in detecting direction for $\left(x_{h},{\dot{x}}_{h}\right)$ = (0,0).

Because Equation (17) satisfies 0<β<1, static pull-in does not occur. Instead, there is a potential well surrounded by heteroclinic orbits, as can be seen in Figure 2. For a fixed value of *R*, the heteroclinic orbits of the unperturbed system (12) can be observed in a plane parallel to the y-O-v plane. Here the black dots in Figure 2 represent saddle points. For *R* = 0, the projection of the heteroclinic orbits can be observed intuitively in the y-O-v plane (see Figure 2b). The sensitive mass undergoes a free stable oscillation in the detecting direction if its initial state is disturbed from the center and within the surroundings of the heteroclinic orbits (see the thick closed loop in Figure 2b). However, if its initial conditions are chosen outside of these surroundings, it undergoes an unbounded motion, as shown in the open loop trajectories in Figure 2b, implying dynamic pull-in. In other words, whether the phenomenon of pull-in occurs depends on the initial conditions of system (11). Therefore, the initial sensitivity for dynamic pull-in, namely pull-in instability, can be discussed only if Equation (9) is satisfied.

## 3. Periodic Responses and Jump of the MEMS Gyroscope Vibrating System

### 3.1. Multiple Periodic Responses

To investigate the jump among coexisting responses, the periodic solution of the non-dimensional system (11) and its local bifurcation should be discussed first, as the practical performance of the micro gyroscope depends on its periodic response. The Method of Multiple Scales (MMS) is applied to determine the periodic solutions approximately. Because the displacement *y* of system (11) is less than 1, for the convenience of applying MMS, we expand the fractional term $\frac{\beta y}{{\left(1-y^{2}\right)}^{2}}$ in Taylor’s series to the three orders of *y*. Then, Equation (11) approximately becomes

(18) $$\frac{d^{2}\tilde{x}}{dT^{2}}+\omega_{1}{}^{2}\tilde{x}=-\mu_{1}\frac{d\tilde{x}}{dT}-k_{1}(3x_{c}{\tilde{x}}^{2}+{\tilde{x}}^{3})+2\eta \frac{dy}{dT}+f\cos\omega T,\;\frac{d^{2}y}{dT^{2}}+\omega_{2}{}^{2}y=-\mu_{2}\frac{dy}{dT}-2\eta \frac{d\tilde{x}}{dT}+Qy^{3},$$

where

(19) $$\omega_{1}{}^{2}=3k_{1}x_{c}{}^{2}+\omega_{x}{}^{2},\;\omega_{2}{}^{2}=1-\beta ,\;Q=2\beta -k_{2}.$$

As the calculated values of the non-dimensional parameters $\mu_{1},\mu_{2},k_{1},k_{2},\eta ,f$ and *Q* in Equation (17) are small, we rescale them in system (18) as

(20) $$\mu_{1}=\varepsilon^{2}{\tilde{\mu }}_{1},\mu_{2}=\varepsilon^{2}{\tilde{\mu }}_{2},k_{1}=\varepsilon^{2}{\tilde{k}}_{1},k_{2}=\varepsilon^{2}{\tilde{k}}_{2},\eta =\varepsilon^{2}\tilde{\eta },f=\varepsilon^{2}\tilde{f},Q=\varepsilon \tilde{Q},$$

where ε is introduced as a small nondimensional parameter. Then, Equation (18) can be rewritten as

(21) $$\begin{array}{l} \frac{d^{2}\tilde{x}}{dT^{2}}+\omega_{1}{}^{2}\tilde{x}=-\varepsilon^{2}{\tilde{\mu }}_{1}\frac{d\tilde{x}}{dT}-3\varepsilon^{2}{\tilde{k}}_{1}x_{c}{\tilde{x}}^{2}-\varepsilon^{2}{\tilde{k}}_{1}{\tilde{x}}^{3}+2\varepsilon^{2}\tilde{\eta }\frac{dy}{dT}+\varepsilon^{2}\tilde{f}\cos(\omega T),\\ \frac{d^{2}y}{dT^{2}}+\omega_{2}{}^{2}y=-\varepsilon^{2}{\tilde{\mu }}_{2}\frac{dy}{dT}+\varepsilon \tilde{Q}y^{3}-2\varepsilon^{2}\tilde{\eta }\frac{d\tilde{x}}{dT}. \end{array}$$

Considering the periodic vibration of the system near the primary resonance and the 1:1 internal resonance of the above system, we set ω in the vicinity of 1 and

(22) $$\omega_{1}=\omega +\varepsilon \sigma_{1},\omega_{2}=\omega +\varepsilon \sigma_{2}.$$

Here, $\sigma_{1}$ and $\sigma_{2}$ are the detuning parameters. On this basis, we rescale $\tilde{x}$,$\tilde{y}$, nondimensional time variable *T* and differential operators as

(23) $$\begin{array}{l} \tilde{x}={\tilde{x}}_{0}+\varepsilon {\tilde{x}}_{1}+\varepsilon^{2}{\tilde{x}}_{2}+\cdots ,\;y=\varepsilon y_{1}+\varepsilon^{2}y_{2}+\varepsilon^{3}y_{3}+\cdots ,\\ T_{0}=\varepsilon^{0}T,T_{1}=\varepsilon^{1}T,T_{2}=\varepsilon^{2}T,T_{3}=\varepsilon^{3}T,\cdots \\ D_{0}=\frac{d}{dT_{0}},D_{1}=\frac{d}{dT_{1}},D_{2}=\frac{d}{dT_{2}},D_{3}=\frac{d}{dT_{3}},\cdots \\ \frac{d}{dT}=D_{0}+\varepsilon D_{1}+\varepsilon^{2}D_{2}+\varepsilon^{3}D_{3}+\cdots . \end{array}$$

By substituting Equations (22) and (23) into Equation (21) and comparing its coefficients of *ε*^0^, *ε*^1^, *ε*^2^ and *ε*^3^, respectively, we have

(24) $$\varepsilon^{0}:D_{0}{}^{2}{\tilde{x}}_{0}+\omega^{2}{\tilde{x}}_{0}=0,$$

(25) $$\varepsilon^{1}:D_{0}{}^{2}{\tilde{x}}_{1}+\omega^{2}{\tilde{x}}_{1}=-2D_{1}D_{0}{\tilde{x}}_{0}-2\omega \sigma_{1}{\tilde{x}}_{0},\;D_{0}{}^{2}y_{1}+\omega^{2}y_{1}=0,$$

(26) $$\begin{array}{cc} \varepsilon^{2}: & D_{0}{}^{2}{\tilde{x}}_{2}+\omega^{2}{\tilde{x}}_{2}=-D_{1}{}^{2}{\tilde{x}}_{0}-2D_{2}D_{0}{\tilde{x}}_{0}-2D_{1}D_{0}{\tilde{x}}_{1}-{\tilde{k}}_{1}{\tilde{x}}_{0}{}^{3}-3{\tilde{k}}_{1}x_{c}{\tilde{x}}_{0}{}^{2}-{\tilde{\mu }}_{1}D_{0}{\tilde{x}}_{0}-2\omega {\tilde{x}}_{1}\sigma_{1}-{\tilde{x}}_{0}\sigma_{1}{}^{2}+\tilde{f}\cos\omega T,\\ & D_{0}{}^{2}y_{2}+\omega^{2}y_{2}=-2D_{1}D_{0}y_{1}-2\tilde{\eta }D_{0}{\tilde{x}}_{0}-2\sigma_{2}\omega y_{1} \end{array}$$

and

(27) $$\varepsilon^{3}:\;\;\;D_{0}{}^{2}{\tilde{y}}_{3}+\omega^{2}{\tilde{y}}_{3}=-D_{1}{}^{2}{\tilde{y}}_{1}-2D_{2}D_{0}{\tilde{y}}_{1}-2D_{1}D_{0}{\tilde{y}}_{2}-2\tilde{\eta }(D_{1}{\tilde{x}}_{0}+D_{0}{\tilde{x}}_{1})-{\tilde{\mu }}_{2}D_{0}{\tilde{y}}_{1}-\sigma_{2}{}^{2}{\tilde{y}}_{1}-2\sigma_{2}\omega {\tilde{y}}_{2}.$$

The solution of Equation (24) can be written as

(28) $${\tilde{x}}_{0}=A(T_{1},T_{2})e^{i\omega T_{0}}+\bar{A}(T_{1},T_{2})e^{-i\omega T_{0}}.$$

where

(29) $$A=\frac{a(T_{1},T_{2})}{2}e^{i\theta (T_{1},T_{2})}.$$

Similarly, the solution $y_{1}$ of Equation (25) can be expressed as

(30) $${\tilde{y}}_{1}=B(T_{1},T_{2})e^{i\omega T_{0}}+\bar{B}(T_{1},T_{2})e^{-i\omega T_{0}}.$$

where

(31) $$B=\frac{b(T_{1},T_{2})}{2}e^{i\varphi (T_{1},T_{2})}.$$

Substituting solutions ${\tilde{x}}_{0}$ and $y_{1}$ into Equation (25) and separating the secular terms yield

(32) $$D_{1}A=i\sigma_{1}A,$$

and

(33) $${\tilde{x}}_{1}=0.$$

Then, by substituting Equations (28)–(33) into Equation (26) and separating the secular terms, we have

(34) $$D_{2}A=\frac{-{\tilde{\mu }}_{1}A}{2}-\frac{\tilde{if}}{4\omega }+\frac{3{\tilde{k}}_{1}A^{2}\bar{A}i}{2\omega },\;D_{1}B=-\tilde{\eta }A+i\sigma_{2}B,$$

and

(35) $${\tilde{x}}_{2}=-\frac{3{\tilde{k}}_{1}x_{c}A\bar{A}}{\omega^{2}}+\frac{{\tilde{k}}_{1}x_{c}A^{2}e^{2i\omega T_{0}}}{\omega^{2}}+\frac{{\tilde{k}}_{1}A^{3}e^{3i\omega T_{0}}}{8\omega^{2}}+cc,\;y_{2}=0.$$

Substituting Equations (32), (34) and (35) into Equation (27) and separating the secular terms there yield

(36) $$D_{2}B=\frac{\tilde{\eta }(\sigma_{2}-\sigma_{1})A}{2\omega }-\frac{{\tilde{\mu }}_{2}B}{2},\;y_{3}=0.$$

Letting $\hat{b}=\varepsilon b$, the periodic solutions of the equivalent nondimensional system (5) in the vicinity of the nontrivial equilibria ($\pm x_{c}$, 0, 0, 0) can be expressed as

(37) $$x=\pm x_{c}+a\cos(\omega T+\theta ),\;y=\hat{b}\cos(\omega T+\varphi ).$$

Considering (29), (31) and

(38) $$\frac{d(\;)}{dT}\approx D_{0}(\;)+\varepsilon D_{1}(\;)+\varepsilon^{2}D_{2}(\;)$$

in Equations (32), (34) and (36), we can obtain

(39) $$\begin{array}{l} \frac{da}{dT}=-\frac{\mu_{1}a}{2}-\frac{f\sin\theta }{2\omega },\\ a\frac{d\theta }{dT}=(\omega_{1}-\omega )a+\frac{3k_{1}a^{3}}{8\omega }-\frac{f\cos\theta }{2\omega },\\ \frac{d\hat{b}}{dT}=\frac{(\omega_{2}-\omega_{1}-2\omega )\eta a}{2\omega }\cos(\theta -\varphi )-\frac{\mu_{2}\hat{b}}{2},\\ \hat{b}\frac{d\varphi }{dT}=\frac{(\omega_{2}-\omega_{1}-2\omega )\eta a}{2\omega }\sin(\theta -\varphi )+(\omega_{2}-\omega )\hat{b}. \end{array}$$

For the periodic solution expressed by Equation (37), the right side of Equation (39) should be zero, yielding

(40) $$\begin{array}{l} \frac{\mu_{1}a}{2}=-\frac{f\sin\theta }{2\omega },\\ (\omega_{1}-\omega )a+\frac{3k_{1}a^{3}}{8\omega }=\frac{f\cos\theta }{2\omega },\\ \frac{\mu_{2}\hat{b}}{2}=\frac{(\omega_{2}-\omega_{1}-2\omega )\eta a}{2\omega }\cos(\theta -\varphi ),\\ (\omega_{2}-\omega )\hat{b}=-\frac{(\omega_{2}-\omega_{1}-2\omega )\eta a}{2\omega }\sin(\theta -\varphi ). \end{array}$$

By eliminating the triangulation function of Equation (40) and returning parameters *f* and η into the original parameters based on Equation (4), we can solve the amplitudes of the periodic solutions in the driving and detecting modes from the following equation:

(41) $$\mu_{1}{}^{2}\omega^{2}a^{2}+(2\omega (\omega_{1}-\omega )a+\frac{3k_{1}a^{3}}{4}{)}^{2}=\frac{16n^{2}\zeta^{2}d_{a}{}^{2}U_{d0}{}^{2}U_{da}{}^{2}}{k_{Y1}{}^{2}d_{eg}{}^{2}s_{d}{}^{2}},\;\hat{b}=\frac{\Omega_{z}a(\omega_{1}+2\omega -\omega_{2})}{\omega_{0}\omega \sqrt{\mu_{2}{}^{2}+4{\left(\omega_{2}-\omega \right)}^{2}}}.$$

As the detected angular velocity $\Omega_{z}$ is independent from $\mu_{2},\;\omega ,\;\omega_{0},\;\omega_{1},\;\omega_{2}$ and *a*, according to the above equation, under a small nonlinear stiffness of the flexure spring in the detecting direction, the response amplitude of the detecting direction, $\hat{b}$, is proportional to the angular velocity of the carrier $\Omega_{z}$, providing the basis for the normal performance of the micro gyroscope. The detection sensitivity *S* = $\frac{\hat{b}}{\Omega_{z}}$ can be given by

(42) $$S=\frac{a(\omega_{1}+2\omega -\omega_{2})}{\omega_{0}\sqrt{\mu_{2}{}^{2}+4{\left(\omega_{2}-\omega \right)}^{2}}\omega }$$

Accordingly, the detection sensitivity *S* increases with *a*, namely the amplitude of the periodic response of the driving mode, which is similar to the linear vibrating system of micro gyroscopes. Nevertheless, as can be seen in Equation (41), due to the occurrence of the nonlinear stiffness $k_{1}$, Equation (41) may have multiple solutions, implying the uncertainty of the gyroscope output. Hence, it is not necessarily the case that the response amplitude of the driving mode a and the detection sensitivity *S* increase with the driving AC voltage amplitude $U_{da}$.

The stability of periodic solutions can be determined with the real parts of the solutions of the characteristic equation of Equation (39), as follows:

(43) $$(\lambda^{2}+\mu_{1}\lambda +\frac{4n^{4}\zeta^{4}{d_{a}}^{2}{U_{d0}}^{2}{U_{da}}^{2}}{{k_{Y1}}^{2}{d_{eg}}^{2}{s_{d}}^{2}\omega^{2}a^{2}}+(\omega_{1}-\omega +\frac{3k_{1}a^{2}}{8\omega })\frac{3k_{1}a^{2}}{4\omega }\left(\lambda^{2}+\mu_{2}\lambda +\frac{{\left(\omega_{2}-\omega_{1}-2\omega \right)}_{2}\eta^{2}a^{2}}{4\omega^{2}{\hat{b}}^{2}}\right)=0$$

In the case of 1:1 internal resonance and primary resonance, we have $\omega_{2}-\omega_{1}-2\omega \neq 0$. Accordingly, there is no pure imaginary or positive-real-part solution for the equation, $\lambda^{2}+\mu_{2}\lambda +\frac{{\left(\omega_{2}-\omega_{1}-2\omega \right)}^{2}\eta^{2}a^{2}}{16\omega^{2}{\hat{b}}^{2}}=0$. It can be concluded that, in Equation (43), the saddle-node bifurcation of the periodic solution occurs only if

(44) $$\frac{4n^{2}\zeta^{2}{d_{a}}^{2}{U_{d0}}^{2}{U_{da}}^{2}}{{k_{Y1}}^{2}{d_{eg}}^{2}{s_{d}}^{2}\omega^{2}a^{2}}+(\omega_{1}-\omega +\frac{3k_{1}a^{2}}{8\omega })\frac{3k_{1}a^{2}}{4\omega }=0$$

**Figure 3:**
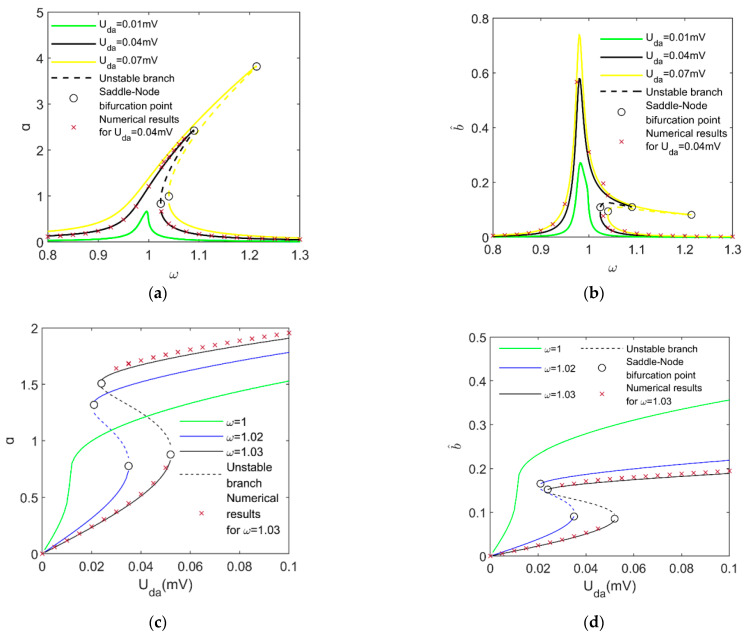
Variation in the amplitudes of periodic solutions in two directions with the driving AC voltage: (**a**) Frequency response in the driving direction; (**b**) Frequency response in the detecting direction; (**c**) Amplitude of driving AC voltage versus magnitude of the driving direction; (**d**) Amplitude of driving AC voltage versus magnitude of the detecting direction.

The variation in the responses with AC voltage is illustrated in Figure 3. The frequency response for the driving and detecting directions are shown in Figure 3a,b, and the changes in the amplitudes of the two directions with the driving AC voltage amplitude are depicted in Figure 3c,d. The solid curves and dashing ones represent stable periodic branches and unstable ones, respectively. Based on Equation (44), the saddle-node points separating the solid and dashing curves are presented. The numerical results for the periodic responses are presented by utilizing the fourth Runge–Kutta approach via MATLAB. For the ordinary differential Equation (5), the software package ODE45 is applied. As indicated in Figure 3, there is good agreement between the analytical prediction and numerical simulations, implying the accuracy of the approximation of the analytical solutions via MMS.

Furthermore, it follows from Figure 3a,b that, at $U_{da}=0.04\mathrm{mV}$ or $U_{da}=0.07\mathrm{mV}$, the frequency response branches bend to the right, yielding multiple stable periodic solutions. This indicates that the coexistence of two periodic responses can be triggered by an increase in the driving AC voltage amplitude when the AC voltage frequency is within the range between the AC voltage frequencies for the two saddle-node bifurcation points.

As depicted by each periodic solution branch of Figure 3c,d, the amplitudes of the periodic responses of the driving and detecting modes increase with the driving AC voltage. Note that there is a jump from the lower solution branch to the higher one when $U_{da}$ increases to its critical value for the right saddle-node bifurcation point (see the branches in the vicinities of the saddle-node bifurcation points).

### 3.2. Description of Jump via Basin of Attraction

To achieve a better display of multistability and the corresponding jump, we apply the fourth Runge–Kutta approach and the cell-mapping method [23,25,28] to display the long-term responses and their basins of attraction (BAs) in the non-dimensional system (5) under primary resonance and 1:1 internal resonance. Note that the initial-condition space of system (5) is four-dimensional, meaning that it is impossible to observe the BAs intuitively. To solve this issue, in this paper, we emphasize that the BAs that we focus on are not the real four-dimensional BAs but their two-dimensional sections, i.e., the projections of BAs in the driving direction x-O-u and the detecting direction y-O-v. When considering the BAs of the driving mode or the detecting one, we assume y(0)=0,v(0)=0 or $x(0)=x_{c},u(0)=0$, respectively. The time step is taken as 1/10^4^ of the period of excitation. To investigate the long-term dynamic behaviors, we assume that an initial condition is safe if the vibration in this initial condition keeps satisfying |*y*(*T*)| < 1 within 10^3^ excited circles; otherwise, it is within the BA of dynamic pull-in, thus being marked in white. Initial conditions that lead to the same periodic response are marked by the same color on the initial plane, as they constitute the BA of an attractor. The colors blue and red are used to mark the BAs of the higher-amplitude periodic response and the lower one, respectively. The BAs are drawn in a sufficiently large space, defined as −3.0 ≤ *x*(0) ≤ 3.0, −3.0 ≤ *u*(0) ≤ 3.0 and −1.0 ≤ *y*(0) ≤ 1.0, −1.0 ≤ *v*(0) ≤ 1.0 in the driving state and detecting state initial planes, respectively. A 160 × 100 array of initial points is generated in each initial plane for each initial state.

**Figure 4:**
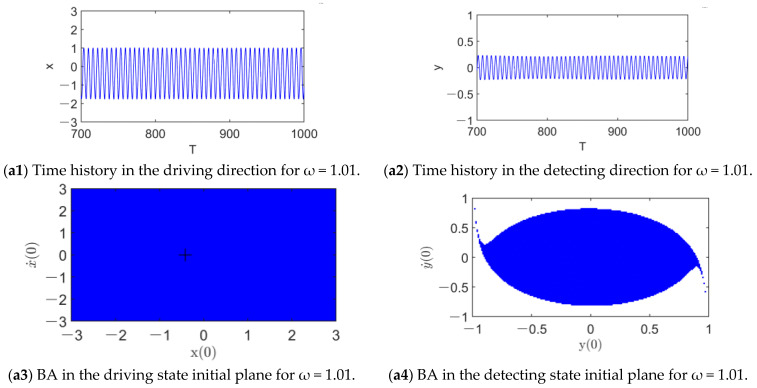

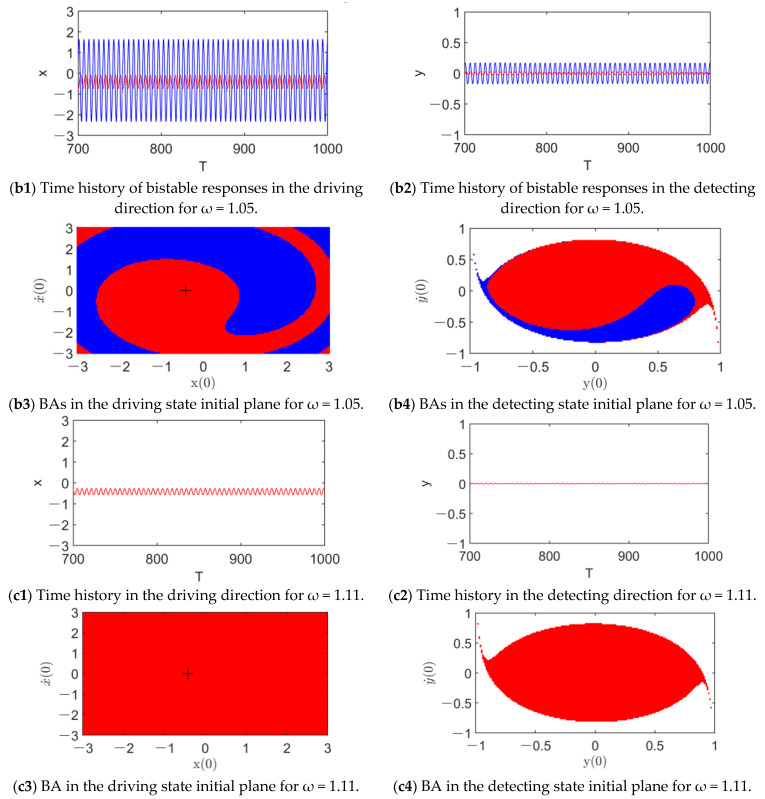
Sequences of responses and their basins of attraction of system (5) with the increase in ω for *U*_da_ = 0.04 mV.

The sequences of responses and their BAs with the increase in the nondimensional AC voltage frequency *ω* are depicted in Figure 4, where the amplitude of the driving AC voltage is fixed as 0.04 mv. For ω = 1.01, there is only one stable periodic response (see Figure 4(a1,a2)). In the driving direction, the whole initial plane is its BA, meaning its global stability, as shown in Figure 4(a3). In contrast, in the detecting direction, there is not only the BA of the periodic response but also the BA of dynamic pull-in (see Figure 4(a4)). Fortunately, the boundary of the BA of the single periodic attractor is smooth, implying that pull-in instability does not occur. As ω increases to 1.05, there are bistable periodic responses (see Figure 4(b1–b4)). This matches the theoretical prediction of the last section well. In addition to the former one, there is a new periodic response with a much lower amplitude. In the vicinity of the equilibrium position ($x_{c},0,0,0$) (see the ‘+’ sign in Figure 4(b3)), the initial conditions lead to the lower-amplitude response rather than the former one, implying the jump phenomenon that the parameter ω induces. As ω reaches 1.11 (see Figure 4(c1–c4), the higher-amplitude response disappears. The lower-amplitude one becomes globally attractive in the driving state plane and coexists with dynamic pull-in in the detecting state plane with a smooth boundary of the BA. It follows from Figure 4 that, with the increase in *ω* in the vicinity of 1, the dynamic behavior of system (5) is changed dramatically. Via disturbing the initial conditions from the equilibrium position ($x_{c},0,0,0$) and increasing the AC voltage frequency, the jump phenomenon can be easily observed. 

**Figure 5:**
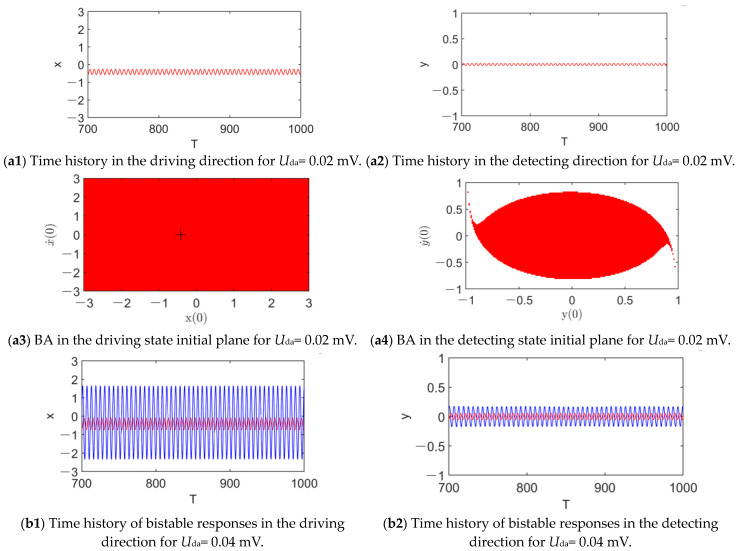

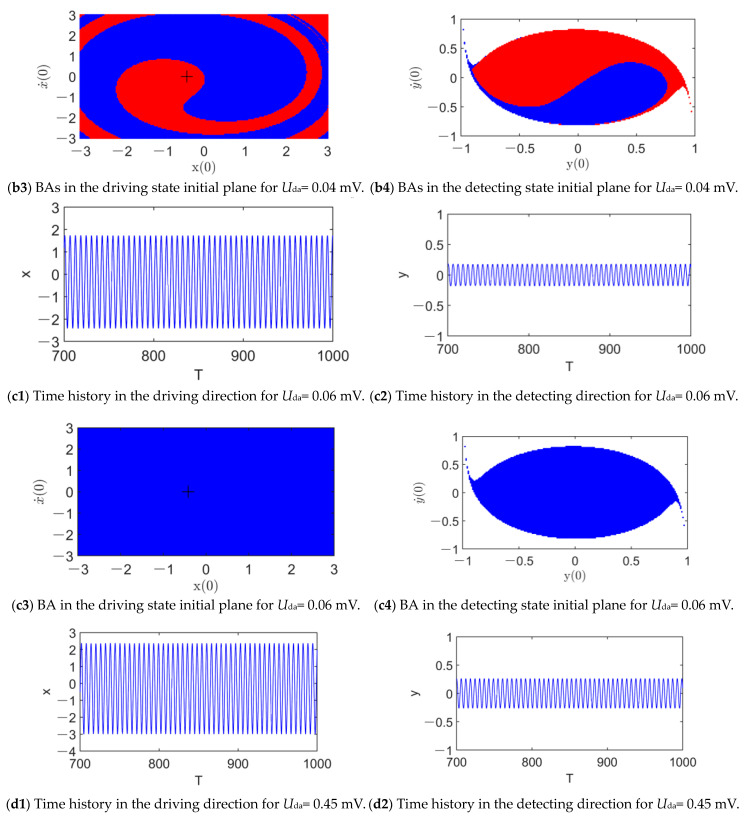

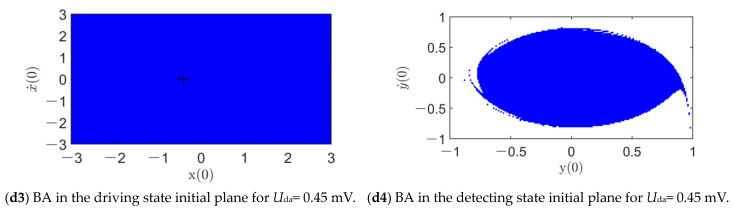
Sequences of dynamic behaviors and their BAs with the increase in U_da_ for ω = 1.03.

The evolution of responses and their BAs with the increase in the driving AC voltage amplitude *U*_da_ is presented in Figure 5, where ω = 1.03. For *U*_da_= 0.02 mV, there is a periodic response (see Figure 5(a1,a2)). As predicted in Figure 3c,d, it comes from the lower amplitude branch, thus being marked in red. In the driving state initial plane, it is globally stable, as can be observed from the red plane in Figure 5(a3). Comparatively, in the detecting state initial plane, it coexists with dynamic pull-in (see Figure 5(a4)). In addition, the smooth boundary of the BA indicates that pull-in instability is not triggered. As *U*_da_ reaches 0.04 mV, a new periodic response appears. According to the analysis of Figure 3c,d, it is from the higher-amplitude branch induced by the saddle-node bifurcation of periodic solutions. As can be observed in Figure 5(b1,b2), its amplitude is much higher than that of the red one. The BAs of the bistable responses are tangled with smooth basin boundaries (see Figure 5(b3,b4)). It is worth mentioning that, in the vicinity of the equilibrium point ($x_{c},0,0,0$), their BAs still coexist, implying that a jump between the two responses can be easily induced by a small disturbance of the initial conditions. For *U*_da_ = 0.06 mV, the lower-amplitude response vanishes. Instead, the higher-amplitude one becomes globally attractive and stable in the driving state initial plane (see Figure 5(c1–c4)). Even though it coexists with dynamic pull-in in the detecting state initial plane, its BA is smooth and occupies a large area around the initial equilibrium point. It is a satisfactory result, as the higher-amplitude periodic response is stable and reliable. According to Equation (42), a stable higher-amplitude periodic response indicates a higher detecting sensitivity. Then, as *U*_da_ increases to 0.45 mV, even though the amplitude of the response in the detecting direction continues to increase, the boundary of its BA becomes fractal, as shown in Figure 5(d2,d4). This implies that the BAs of the periodic response and dynamic pull-in mix with each other, thus triggering pull-in instability.

## 4. Pull-in Instability of the MEMS Gyroscope

In this section, necessary conditions and numerical simulations for pull-in instability are presented. The vibration of the nondimensional system (5) is the so-called dynamic pull-in if its amplitude exceeds 1 at a certain moment, demonstrating an unbounded solution escaping from the potential well. The union of all corresponding initial conditions, i.e., the BA of dynamic pull-in, is unsafe for the work performance of the micro gyroscope. Therefore, the union of the BAs of all the bounded responses is the so-called safe basin [23,26]. Pull-in instability can be described by the erosion of the safe basin [27,28], namely the fractality of safe basin boundaries, meaning that a tiny disturbance of initial conditions induces an excessive or unbounded vibration. Because pull-in instability indicates the loss of the global integrity of the MEMS gyroscope system, it is usually attributed to global bifurcation. Based on the analysis of Section 2, there are heteroclinic orbits in the unperturbed system of system (5) depicted in three-dimensional space. The critical conditions for the heteroclinic tangency of the stable and unstable manifolds can be analyzed in the three-dimensional space *R-y-v*. The Melnikov method, a typical method used for discussing global bifurcations [29], is utilized to predict the necessary conditions for pull-in instability in the detecting direction of the micro gyroscope.

According to the heteroclinic orbits shown in Figure 2a, by fixing the value of *R* in the three-dimensional space *y-v-R*, we may have a two-dimensional section heteroclinic orbits $\left(y_{h},\pm v_{h}\right)$, similar to that in Figure 2b. Based on Equation (16), $y_{h}$ and $v_{h}$ are functions of *R*. For each section parallel to the *y-o-v* plane, the heteroclinic orbits cross two saddle points, noted by ${\hat{S}}_{\pm }(0,R,\pm \hat{y},0)$, where

(45) $$R^{2}+{\hat{y}}^{2}+\frac{k_{2}{\hat{y}}^{4}}{2}-\frac{\beta }{1-{\hat{y}}^{2}}=y_{s}{}^{2}+\frac{k_{2}y_{s}{}^{4}}{2}-\frac{\beta }{1-y_{s}{}^{2}}.$$

According to the condition $H(\frac{R_{\max}}{\omega_{x}},0,0,0)=H(0,0,y_{s},0)$, the maximum value of the parameter R noted by $R_{\max}$ can be determined below:

(46) $$R_{\max}=y_{s}\sqrt{\frac{1-\beta -y_{s}{}^{2}}{1-y_{s}{}^{2}}}.$$

As the heteroclinic orbits $\left(y_{h},\pm v_{h},R\right)$ cannot be expressed by the explicit functions of the time *T*, we cannot employ the Melnikov function directly. To deal with this problem, we introduce a new transformation parameter ϕ to express the heteroclinic orbits and the time *T* as the explicit functions of ϕ. It is assumed that [30]

(47) $${\phi |}_{T=-\infty }=0,\;{\phi |}_{T=0}=\frac{\pi }{2},\;{\phi |}_{T=+\infty }=\pi ,\;\frac{d\phi }{dT}=\Phi (\phi ),\;\Phi (\phi +2\pi )=\Phi (\phi ).$$

The heteroclinic orbits can be expressed as

(48) $$y_{h}=\hat{y}\cos\phi ,\;v_{h}=-\Phi \hat{y}\sin\phi$$

Substituting Equations (47) and (48) into the unperturbed system (12) yields

(49) $$\frac{1}{2}v^{2}=\frac{1}{2}{\left(-\Phi \hat{y}\sin\phi \right)}^{2}=\int_{\hat{y}}^{y}(-y-k_{2}y^{3}+\frac{\beta y}{{\left(1-y^{2}\right)}^{2}})dy$$

Hence, we have

(50) $$\Phi =\sqrt{1+\frac{k_{2}{\hat{y}}^{2}}{2}(1+\cos^{2}\phi )-\frac{\beta }{\left(1-{\hat{y}}^{2}\cos^{2}\phi )(1-{\hat{y}}^{2}\right)}}$$

Then, substituting Equation (50) into Equation (47) and integrating it yield the following explicit function of the time *T*:

(51) $$T=-\frac{\sqrt{2}\mathrm{sign}(\cos\phi )(1-{\hat{y}}^{2})}{\hat{y}P_{1}}({\hat{y}}^{2}F_{1}(\arcsin(\hat{y}\left|\cos\phi \right|),-P_{2})+(1-{\hat{y}}^{2})F_{3}(\frac{1}{{\hat{y}}^{2}},\arcsin(\hat{y}\left|\cos\phi \right|),-P_{2}))$$

where $P_{1}=\sqrt{{\left(1-{\hat{y}}^{2}\right)}^{2}+\beta (2{\hat{y}}^{2}-1)},P_{2}=\frac{\beta -{\left(1-{\hat{y}}^{2}\right)}^{2}}{P_{1}^{2}}$.

To apply the Melnikov method, we first rewrite the non-dimensional system (11) as

(52) $$\frac{d\tilde{x}}{dT}=u+g^{\tilde{x}},\;\frac{du}{dT}=-\omega_{x}{}^{2}\tilde{x}+g^{u},\;\frac{dy}{dT}=v+g^{y},\;\frac{dv}{dT}=-y-k_{2}y^{3}+\frac{\beta y}{{\left(1-y^{2}\right)}^{2}}+g^{v},$$

where

(53) $$g^{\tilde{x}}=0,\;g^{u}=-\mu_{1}u-k_{1}(3x_{c}{}^{2}\tilde{x}+3x_{c}{\tilde{x}}^{2}+{\tilde{x}}^{3})+\eta v+f\cos\omega T,\;g^{y}=0,\;g^{v}=-\mu_{2}v-\eta u.$$

Then, substituting the functions of the heteroclinic orbits and the time *T* into the Melnikov integrals of system (11), we have

(54) $$\begin{array}{ll} M_{1} & =\int_{-\infty }^{+\infty }{\left(xg^{x}+ug^{u}\right)|}_{\left(x_{h},u_{h},y_{h},\pm v_{h}\right)}dT\\ & =-\mu_{1}R^{2}(\frac{\pi }{2}-\frac{\sin(2\omega_{x}\pi )}{4\omega_{x}})-2\eta R\hat{y}I_{2}\pm fRE_{0}\sin(\omega T_{0}-\gamma ), \end{array}$$

and

(55) $$\begin{array}{ll} M_{2} & =\int_{-\infty }^{+\infty }\left((y+k_{2}y^{3}-\frac{\beta y}{{\left(1-y^{2}\right)}^{2}})g^{y}+vg^{v}\right)dT\\ & =\int_{-\infty }^{+\infty }(-\mu_{2}v_{h}-2\eta u_{h})v_{h}dT\\ & =-\mu_{2}{\hat{y}}^{2}I_{1}+2\eta R\hat{y}I_{2} \end{array}$$

where

(56) $$\begin{array}{l} I_{1}=\int_{0}^{\pi }(\Phi (\phi )\sin^{2}\phi )d\phi ,\;I_{2}=\int_{0}^{\pi }\sin(\omega_{x}T(\phi ))\sin\phi d\phi ,\\ E_{0}=\sqrt{\left(\frac{\sin^{2}\frac{(\omega_{x}-\omega )\pi }{2}}{{\left(\omega_{x}-\omega \right)}^{2}}+\frac{\sin^{2}\frac{(\omega_{x}+\omega )\pi }{2}}{{\left(\omega_{x}+\omega \right)}^{2}})+\frac{\cos(\omega_{x}\pi )-\cos(\omega \pi )}{\left(\omega_{x}{}^{2}-\omega^{2}\right)}\cos(\omega \pi \right)},\\ \gamma =\arccos(\frac{\sin\frac{(\omega_{x}-\omega )\pi }{2}}{(\omega_{x}-\omega )E_{0}}\cos\frac{(\omega_{x}-\omega )\pi }{2}-\frac{\sin\frac{(\omega_{x}+\omega )\pi }{2}}{(\omega_{x}+\omega )E_{0}}\cos\frac{(\omega_{x}+\omega )\pi }{2}). \end{array}$$

When $\omega_{x}=\omega =1$, according to the limits of the above equation, we have $E_{0}=\frac{\pi }{2},\gamma =0$.

For the occurrence of the transverse intersection between the stable and unstable heteroclinic manifolds of system (11), there should be a simple zero in the Melnikov functions (53) and (54). Letting the function *M*_2_ be zero, we can solve *R* in the following equation:

(57) $$2\eta RI_{2}-\mu_{2}\hat{y}I_{1}=0$$

Substituting Equations (54) and (55) into *M*_1_ = 0, we conclude that the Melnikov function can have a simple equilibrium only if

(58) $$f\geq \frac{\mu_{1}R^{2}(2\omega_{x}\pi -\sin(2\omega_{x}\pi ))+4\mu_{2}\omega_{x}{\hat{y}}^{2}I_{1}}{4\omega_{x}RE_{0}}$$

Expressing the nondimensional parameter *f* using the original system parameters, we can obtain the necessary condition of the driving AC voltage amplitude for heteroclinic bifurcation, given by

(59) $$U_{da}\geq \frac{k_{X1}d_{eg}s_{d}}{16\omega_{x}RE_{0}n\zeta d_{a}U_{d0}}(\mu_{1}R^{2}(2\omega_{x}\pi -\sin(2\omega_{x}\pi ))+4\mu_{2}\omega_{x}{\hat{y}}^{2}I_{1}).$$

It follows that the increase in the amplitude of the driving AC voltage may lead to pull-in instability of the micro gyroscope.

For the given values of the parameters in Equation (17), the critical value of $U_{da}$ can be calculated as $U_{da}^{0}\approx 0.12\mathrm{mV}$. This shows that pull-in stability can be ensured when $U_{da}$ is less than 0.12 mV. Reviewing the BAs of the detecting state initial plane of Figure 5, we can easily observe that, for $U_{da}$ increasing from 0.02 mV to 0.06 mV, even though the steady responses and their BAs change qualitatively (see Figure 5(a4,b4,c4)), the safe basins of the detecting mode, namely the union of the BAs of bounded attractors, are still similar to smooth boundaries to separate unsafe zones for dynamic pull-in. In contrast, for $U_{da}=0.45\mathrm{mV}$, more than $U_{da}^{0}$, the safe basin of system (11), i.e., the BA of the high-amplitude attractor (see Figure 5(d4)), becomes fractal, indicating that either a large initial displacement or velocity in the detecting direction results in poor pull-in stability. Hence, the evolution of the safe basin of the detecting mode in Figure 5 matches the analytical prediction in this section well. Note that, in Figure 5(d4), despite the fractal basin boundary, no initial conditions in the vicinity of the point (0,0) develop into pull-in, showing a low possibility of pull-in occurrence.

**Figure 6:**
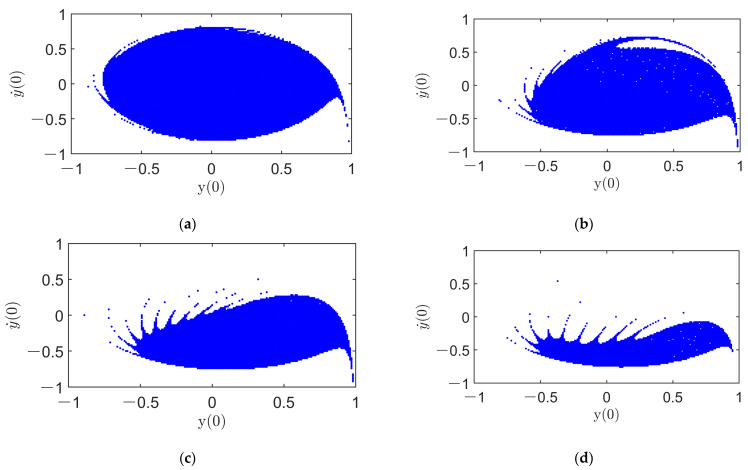
Evolution of safe basins of system (5) in the detecting state initial plane with the increase in *U_da_* for *ω* = 1.03: (**a**) *U*_da_= 0.50 mV; (**b**) *U*_da_= 50 mV; (**c**) *U*_da_= 55 mV; (**d**) *U*_da_= 60 mV.

Now, continuing to increase $U_{da}$, the sequences of the safe basin of system (11) in the detecting state initial plane $y(0)-\dot{y}(0)$ are illustrated in Figure 6. As $U_{da}$ varies from 0.50 mV to 60 mV, there is only one bounded attractor in system (11), namely a periodic response, similar to that of Figure 5(d1,d2)). Hence, a safe basin in the detecting state initial plane is just the BA of the periodic response in the detecting direction. Similar to Figure 5(d4), the safe basin of Figure 6 is marked in blue. As can be seen in Figure 6, with the increase in $U_{da}$, the fractality of the safe basin becomes more and more visible. The safe basin is shrunk toward (0,0) when the driving AC voltage amplitude increases greatly. Consequently, the probability of a pull-in occurrence becomes higher, indicating the aggravation of pull-in instability. Specifically, as shown in Figure 6c,d, the vicinity of (0,0) is eroded, showing pull-in instability and an extremely high possibility of a pull-in occurrence of the micro gyroscope. The sequences of safe basins in Figure 5 and Figure 6 illustrate that, due to the heteroclinic bifurcation of the MEMS gyroscope system, the increase in the amplitude of the driving AC voltage leads to pull-in instability. Therefore, the amplitude of the driving AC voltage should be selected to be a bit lower than the threshold $U_{da}^{0}$ to ensure a single high-amplitude periodic response and to avoid jumps and pull-in instability. According to the quantitative results of Figure 6, at the very least, $U_{da}$ should be less than 10 times $U_{da}^{0}$ to avoid the occurrence of dynamic pull-in in the vicinity of (0,0).

## 5. Discussion

In this paper, the dynamics of a comb-tooth micro gyroscope considering the nonlinearities of its stiffness and electrostatic forces are explored. We focus on the effect of the driving voltage on causing two types of initial-sensitive dynamic behaviors, i.e., jump and pull-in instability. By introducing nondimensional parameters, we obtain the nondimensional system. On this basis, under the primary resonance and 1:1 internal resonance of the system, the variation in periodic responses with the driving AC voltage is investigated via MMS. Basins of attraction are classified to quantitatively depict jumps among coexisting attractors induced by the disturbance of the initial conditions. Furthermore, the theory of global bifurcation is employed to predict the conditions for the pull-in instability of the micro gyroscope. By introducing the erosion of the safe basin to describe pull-in instability intuitively, the cell-mapping approach is applied to present numerical results that are in great agreement with the qualitative results, verifying the accuracy of the prediction. Several significant results are presented as follows.

First, the static bifurcation analysis of the equilibria shows that there is a critical value of the detecting voltage for triggering static pull-in of the micro gyroscope in the detecting direction; thus, the detecting voltage should be lower than it to avoid static pull-in.

Second, when the stiffness nonlinearity in the detecting direction is weak, the output of the micro gyroscope is still proportional to the angular velocity to be measured, which is the basis for the normal performance of the micro gyroscope. However, this does not necessarily mean that the micro gyroscope can perform steadily, as the stiffness nonlinearity in the driving direction can lead to bistable periodic responses and thus the uncertainty of the gyroscope output.

Third, due to the saddle-node bifurcation of the periodic solution, the nonlinear hardening behavior is typical right bending in the driving mode but right-down bending in the detecting mode. This indicates that, when the frequency of the driving AC voltage is within the right-side neighborhood of the natural frequency of the detecting mode, the increase in the driving AC voltage amplitude may induce bistability, and disturbances of the initial conditions may trigger jumps between bistable responses or dynamic pull-in, thus leading to the unreliability or insecurity of the micro gyroscope. Via the classification of basins of attraction, an applicable confident estimate of the system response can be developed to satisfy the stable output response of the gyroscope.

Finally, owing to the heteroclinic bifurcation of the micro gyroscope system, an increase in the driving AC voltage amplitude can lead to the aggravation of pull-in instability and an increasing probability of the occurrence of dynamic pull-in. Hence, the driving AC voltage amplitude should be selected to be a bit lower than the predicted threshold to ensure a steady high-amplitude output and thus a high reliability for the MEMS gyroscope.

## 6. Conclusions

The present work performs an extensive analysis of the jump and pull-in instability of a typical MEMS gyroscope, which not only significantly expands current knowledge of initial-condition-sensitive behaviors but also has a certain guidance for achieving the safe operation and desired outcome of MEMS gyroscopes in practical applications. Because the results are based on the fixed physical properties of the microstructure, the ranges of the applicability of the obtained results as well as the limitations are specified. However, it is worth noting that the approach we use is general for nonlinear micro gyroscope systems. Further carrying out the study of the influence of these physical properties on global dynamics would be beneficial for the optimization of the structure design of MEMS gyroscopes, which will be included in our future work.

The investigation is mainly focused on the range where simulations could have an experimental counterpart. The construction of the experimentation to confirm the predictions of a jump from one attractor to the other one, as well as the disturbance-induced dynamic pull-in of the micro gyroscope, will also be our main concern in the future.

## Figures and Tables

**Table 1:** Parameters of the MEMS gyroscope [15].

Parameter (Unit)	Symbol	Value
Equivalent mass (kg)	*M*	0.14 × 10^−7^
Equivalent damping in the driving direction (N•s/m)	$c_{X}$	1.3517 × 10^−5^
Equivalent damping in the detecting direction (N•s/m)	$c_{Y}$	1.3517 × 10^−5^
Linear stiffness coefficient in the driving direction (N/m)	$k_{X1}$	58
Cubic stiffness coefficient in the driving direction (N/m^3^)	$k_{X2}$	0.12 × 10^12^
Linear stiffness coefficient in the detecting direction (N/m)	$k_{Y1}$	61.053
Cubic stiffness coefficient in the detecting direction (N/m^3^)	$k_{Y2}$	0.12 × 10^12^
Number of comb teeth	n	9
Overlap length of comb teeth in the driving direction (m)	l	2.458 × 10^−4^
Thickness of comb teeth in the driving direction (m)	$d_{a}$	4.916 × 10^−5^
Gap of comb teeth in the driving direction (m)	$s_{d}$	4.916 × 10^−6^
Dielectric constant	ζ	1.0059
Detecting electrode length (m)	$l_{l}$	1.17 × 10^−4^
Detecting electrode width (m)	$l_{w}$	2.9275 × 10^−5^
Detecting electrode clearance (m)	$d_{eg}$	4.916 × 10^−6^
Detecting voltage (V)	$U_{s}$	0.2 × 10^−3^
Acceleration of the proof mass in the driving direction (m/s^2^)	$a_{X}$	8146.5
Acceleration of the proof mass in the detecting direction (m/s^2^)	$a_{Y}$	0
Angular velocity being measured (°/s)	$\Omega_{z}$	200
DC bias voltage in the driving direction (V)	$U_{d0}$	7.8788 × 10^−4^
Amplitude of AC voltage in the driving direction (V)	$U_{da}$	
Frequency of AC voltage in the driving direction (HZ)	Ω	

## Data Availability

Not applicable.
